# Modulation of Fabrication and Nutraceutical Delivery Performance of Ovalbumin-Stabilized Oleogel-Based Nanoemulsions via Complexation with Gum Arabic

**DOI:** 10.3390/foods11131859

**Published:** 2022-06-24

**Authors:** Yuxing Gao, Zihua Wang, Changhu Xue, Zihao Wei

**Affiliations:** 1 College of Food Science and Engineering, Ocean University of China, Qingdao 266003, China; gaoyuxing@stu.ouc.edu.cn (Y.G.); wangzihua@stu.ouc.edu.cn (Z.W.); xuech@ouc.edu.cn (C.X.); 2 Laboratory for Marine Drugs and Bioproducts, Qingdao National Laboratory for Marine Science and Technology, Qingdao 266237, China

**Keywords:** oleogel-based nanoemulsions, protein–polysaccharide complexes, nutraceutical delivery, ovalbumin, astaxanthin

## Abstract

Protein–polysaccharide complexes, which involve Maillard-type protein–polysaccharide conjugates and electrostatic protein–polysaccharide complexes, have the potential to stabilize oleogel-based nanoemulsions for nutraceutical delivery. Here, ovalbumin (OVA) and gum arabic (GA) were used to prepare OVA–GA conjugate (OGC) and OVA–GA mixture (OGM), followed by the fabrication of astaxanthin-loaded oleogel-based nanoemulsions. Carnauba wax (5% *w*/*w*) and rice bran oil were mixed to prepare food-grade oleogel. The successful preparation of OGC was verified by means of SDS-PAGE analysis and free amino groups determination. OGC endowed oleogel-based nanoemulsions with smaller emulsion droplets and higher stability during 30-day storage, implying more outstanding emulsifying capability than OGM. Both OGC-stabilized nanoemulsions and OGM-stabilized nanoemulsions could enhance the extent of lipolysis and the bioaccessibility of astaxanthin compared with oleogel. Meanwhile, OGC exhibited significantly better than OGM, which indicated that OGC-stabilized oleogel-based nanoemulsions possessed more desirable nutraceutical delivery performance than OGM-stabilized oleogel-based nanoemulsions. This study may fill a gap in the influence of different protein–polysaccharide complexes on oleogel-based nanoemulsions and contribute to deeper insights about novel oleogel-based nanoemulsions for their applications in the food industry.

## 1. Introduction

Oleogel-based emulsions have been demonstrated to be the outstanding delivery systems for bioactive ingredients [1,2]. When compared with conventional oil-in-water emulsions (without oleogel), oleogel-based emulsions exhibit better long-term storage stability and freeze-thaw stability [3]. The desirable stability for easy storage and transportation, coupled with remarkable delivery performance of bioactive ingredients, has led to the recent increased scholarly attention on oleogel-based emulsions [4,5,6]. Nanoemulsions with high stability to particle aggregation and gravitational separation can significantly enhance the bioavailability of the encapsulated nutrients [7]. It is essential to design novel oleogel-based nanoemulsions with satisfactory nutraceutical delivery performance for the practical application of functional foods. Ovalbumin (OVA), which is over half of the entire protein in egg albumen, is a globular glycoprotein with an ideal amino acid balance [8]. Our preliminary experiments have indicated that OVA cannot stabilize oleogel-based nanoemulsions very well, although it is generally accepted that OVA possesses satisfactory interfacial and emulsifying properties [9]. There is thus a significant challenge to improve its emulsifying capability. Multiple studies have shown that protein–polysaccharide complexes exhibit superior emulsifying capability and nutrient protection compared with proteins alone [10,11,12]. Both electrostatic protein–polysaccharide complexes and Maillard-type protein–polysaccharide conjugates belong to protein–polysaccharide complexes and can be utilized to stabilize nanoemulsions [13,14,15]. However, the correlation between protein–polysaccharide interactions and the emulsifying capability of the protein–polysaccharide complexes has not yet been systematically investigated. Furthermore, the comparisons between electrostatic protein–polysaccharide complexes and Maillard-type protein–polysaccharide conjugates remain to be explored.

It is commonly known that astaxanthin shows good capability to prevent inflammation, cardiovascular disease and immune response as a bright red secondary carotenoid with outstanding antioxidant activity [16]. Since astaxanthin is poorly bioavailable in the human body as a result of its strong hydrophobicity [17], it is of great importance to enhance astaxanthin bioaccessibility by developing food-grade delivery vehicles. In addition, the feasibility of nanoemulsions for delivering astaxanthin has been confirmed through scientific experiments [18,19]. Therefore, astaxanthin can serve as the model nutrient in the oleogel-based nanoemulsions in this research.

Rice bran, the by-product of rice production possessing approximately 60% of the total nutrients in whole rice grain, can be used to extract rice bran oil through the hydraulic press method or via non-polar solvent extraction [20]. Rice bran oil with favorable antioxidant and anti-inflammatory activity is a relatively healthy oil which contains 33% polyunsaturated fatty acids (PUFAs) and nearly half monounsaturated fatty acids (MUFAs) [21]. Considering that individuals pay more attention to the nutritional value of food products in recent times, rice bran oil can be utilized to exploit functional foods for meeting consumers’ needs. Several studies so far have focused on the development of food-grade emulsions based on rice bran oil which are meaningful for the full utilization of the by-product of rice production [22,23].

Herein, the influence of protein–polysaccharide complexes on the oleogel-based nanoemulsions for enhanced nutraceutical delivery was, to the best of our knowledge, investigated for the first time. This work started with the fabrication of OVA–gum arabic (GA) complexes. The properties of the obtained OVA–GA conjugate (OGC) and OVA–GA mixture (OGM) were then investigated. Subsequently, the oleogel was prepared using rice bran oil and carnauba wax, and the minimum concentration of oleogelator in oleogel was measured. Finally, the oleogel-based nanoemulsions were fabricated by the OGC solution or OGM solution (aqueous phase) and the oleogel (oil phase). After the systematical characterization of the nanoemulsions, in vitro lipolysis and astaxanthin bioaccessibility in astaxanthin-loaded oleogel-based nanoemulsions were investigated. In addition to facilitating the high-value utilization of rice bran oil, the present work may lay the foundation for preparing novel oleogel-based nanoemulsions stabilized by protein–polysaccharide complexes in order to improve nutraceutical delivery.

## 2. Materials and Methods

### 2.1. Materials

Ovalbumin (OVA) was purchased from Ruiyong Biological Technology Co., Ltd. (Shanghai, China). Rice bran oil was purchased from Zhejiang Delekang Food Co., Ltd. (Taizhou, China). Gum arabic (GA), carnauba wax, o-Phthalaldehyde, brilliant blue R, glycine, tris (hydroxymethyl) aminomethane and sodium dodecyl sulfate were purchased from Aladdin Biochemical Technology Co., Ltd. (Shanghai, China). Astaxanthin was purchased from Shanxi Kangsheng Biotechnology Co., Ltd. (Xi’an, China). Sodium tetraborate, 2-Mercaptoethanol, methanol and glacial acetic acid were obtained from Sinopharm Chemical Reagent Co., Ltd. (Shanghai, China). An SDS-PAGE Assay Kit was purchased from Epizyme Biomedical Technology Co., Ltd. (Shanghai, China). The protein marker was purchased from Solarbio Bioscience & Technology Co., Ltd. (Shanghai, China). A UPD-I-10T Ulupure purification system (Chengdu Ultrapure Technology Co., Ltd., Chengdu, China) was applied to produce ultrapure water used throughout the experiment.

### 2.2. Preparation of OVA–GA Complexes Conjugate (OGC)

The OVA–GA mixture (OGM) at pH 7.0 was prepared by dissolving 20 mg/mL OVA and 4 mg/mL GA in ultrapure water prior to freeze-drying. Subsequently, an oven (the relative humidity was 79%) was employed to keep the lyophilized powders at 60 °C for different times of 24 h, 48 h and 96 h. The obtained OVA–GA conjugate (OGC) was stored at −20 °C before application.

### 2.3. Preparation of Carnauba Wax-Based Oleogel

The carnauba wax-based oleogel was prepared based on the method of Wei and Huang [6]. Briefly, carnauba wax was mixed with rice bran oil at different mass ratios (2%, 3%, 4% and 5% *w*/*w*) and stirred at the oil bath of 90 °C until the mixture was totally dissolved. The samples were subsequently inverted at room temperature to observe their fluidity. When the sample could not flow after 1 h of inversion, the corresponding carnauba wax content was regarded as the critical gelling concentration.

### 2.4. Characterization of OGC

#### 2.4.1. Sodium Dodecyl Sulfate Polyacrylamide Gel Electrophoresis (SDS-PAGE)

SDS-PAGE was performed in accordance with the methodology described previously, with some modifications [24]. The samples including native OVA and glycosylated OVA with different heat treatment times (24 h, 48 h and 96 h) were separated on a precast 12.5% acrylamide separating gel under a voltage of 120 V. Coomassie Brilliant Blue R-250 was applied to stain the gel sheet, which was then decolorized and recorded.

#### 2.4.2. Zeta Potential

A Malvern nanoparticle size potentiometer (Zetasizer Nano zs90) was employed to examine the zeta potential of OGC heated for 96 h over the pH range of 2.0 to 9.0, and the OGM at corresponding pH values were measured as controls.

#### 2.4.3. Free Amino Groups

An ortho-phthaldialdehyde (OPA) method was applied to measure the free amino groups of native OVA and OGC, respectively [25]. OPA (80 mg), 0.1 M sodium tetraborate solution (50 mL), methanol (2 mL), 20% *w*/*w* sodium dodecyl sulfate (5 mL) and mercaptoethanol (200 µL) were used to prepare the OPA reagent. Afterwards, 2 mg/mL protein solution (200 µL) and OPA reagent (4 mL) were mixed and then incubated at 35 °C for 2 min. Finally, the absorbance at 340 nm of the samples was examined and the concentration of free amino groups was obtained by means of an L-leucine calibration curve.

### 2.5. Preparation of Oleogel-Based Nanoemulsions

**Figure 1:**
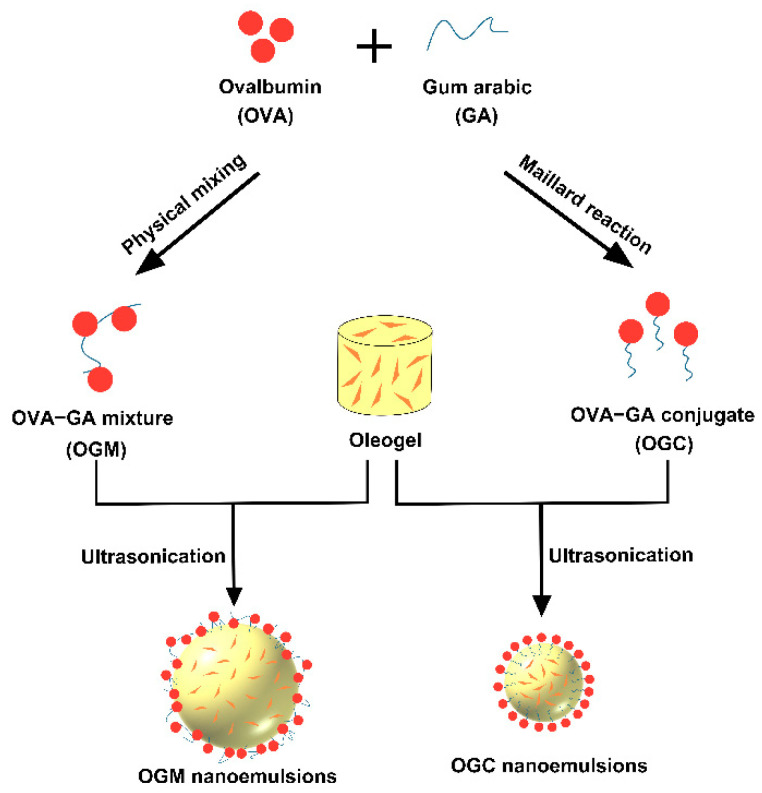
Schematic illustration of the fabrication of oleogel-based nanoemulsions.

Figure 1 shows the preparation process of oleogel-based nanoemulsions. Astaxanthin (0.2% *w*/*w*) was dissolved in the oleogel structured by 5% *w*/*w* carnauba wax under heating to prepare the astaxanthin-loaded oleogel. OGC solutions (20–50 mg/mL) at pH 2.0 were added to the formed oleogel to obtain mixed systems (oil fraction *φ* = 0.9). Subsequently, the mixed systems were sonicated using a SM-900A ultrasonic processor (Nanjing Shunma Instrument Equipment Co., Ltd., Nanjing, China) for 3 min and the frequency was fixed at 20 kHz. The influence of OGC concentrations on the properties of oleogel-based nanoemulsions was also investigated. The same experimental conditions were employed to prepare the oleogel-based nanoemulsions using the OGM solutions instead of the OGC solutions.

### 2.6. Characterization of Oleogel-Based Nanoemulsions

#### 2.6.1. Droplet Size of Oleogel-Based Nanoemulsions

The droplet size of oleogel-based nanoemulsions was measured by means of an Intelligent laser particle sizer (Bettersize 2600, Dandong Bettersize Instruments Ltd., Dandong, China). The samples were diluted 100 times using pH-adjusted ultrapure water (pH 2.0) prior to measurement.

#### 2.6.2. Storage Stability Analysis

The obtained oleogel-based nanoemulsions were stored at a refrigerator temperature of 4 °C. The storage stability of the samples was determined by observing and recording the sample during 30-day storage.

### 2.7. Lipolysis and Bioaccessibility of Astaxanthin in Oleogel-Based Nanoemulsions

#### 2.7.1. Digestion of Oleogel-Based Nanoemulsions

The in vitro gastrointestinal digestion of oleogel-based nanoemulsions was carried out using the method described previously with slight modification [26]. Sodium chloride (2 g) was dissolved in 1 L of pH-preset ultrapure water (pH 1.2) to prepare simulated gastric fluid (SGF). The samples containing 2 g of oleogel were added to SGF (16 mL). The gastric digestion process was started with the addition of SGF (4 mL) containing freshly dissolved pepsin (32 mg). After 120 min at 37.0 ± 0.1 °C, the digestion was terminated by regulating the pH of digesta to 7.5. During the gastric digestion process, CaCl_2_ (10 mM), bile salt (10 mg/mL) and Tris (50 mM) were dissolved to pH-preset ultrapure water (pH 7.5) for obtaining simulated intestinal fluid (SIF). An equal volume of SIF containing pancreatin (3.2 mg/mL) was added to the gastric digesta for initiating the intestinal digestion process. Over the 120-min incubation period at 37.0 ± 0.1 °C, NaOH solution (0.25 M) was used to keep pH at 7.5 and the volume of NaOH was recorded. Thereafter, the samples were centrifuged at 10,000× *g* for 40 min to obtain the clear micelle phase.

The release of free fatty acids (FFA) could serve as the parameter to characterize lipolysis of digestion samples. It is generally accepted that 2 mol of FFA could be released from 1 mol of triglycerides and 2 mol of NaOH was thus consumed. The fraction of FFA released (% FFA) was calculated according to Equation (1) [26]:

(1) $$\%\mathrm{FFA}=100\;\times \;\frac{M_{\text{Rice bran oil }}{\times V}_{\text{NaOH}}{\times m}_{\text{NaOH}}}{w_{\text{Rice bran oil}}\times 2}$$

where $M_{\text{Rice bran oil }}$ was the molecular mass of the rice bran oil (in g/mol), $V_{\text{NaOH}}$ was the volume of NaOH solution (0.25 M) added to the digesta (in L), $m_{\text{NaOH}}$ was the molarity of NaOH (in mol/L), $w_{\text{Rice bran oil}}$ was the total mass of initially present rice bran oil (in g). The average molecular mass of rice bran oil was taken as 864 g/mol [27].

#### 2.7.2. High-Performance Liquid Chromatography (HPLC) Analysis of Astaxanthin

Astaxanthin content in the micelle phase was examined with an Agilent 1260 HPLC system (Agilent Technologies Inc., Santa Clara, CA, USA) with a YMC-C18 (YMC CO., Ltd., Kyoto, Japan) chromatography column (4.6 mm × 250 mm, 5 µm). The HPLC mobile phase was the combination of (A) methanol and (B) methyl tert-butyl ether. The elution condition was set as follows: 0–10 min, 90% A; 10–30 min, 40% A; 30–40 min, 90% A. The flow velocity was set as 1 mL/min and the sample volume was 30 µL. Quantification of astaxanthin according to a standard curve of astaxanthin was performed at a wavelength of 476 nm.

#### 2.7.3. Determination of Astaxanthin Bioaccessibility

Astaxanthin bioaccessibility was calculated by the following equation after HPLC measurement of astaxanthin in the micelle phase:

(2) $$\%\text{bioaccessibility}=\frac{\text{astaxanthin content in the micelle phase}}{\text{total astaxanthin content in theformulations }}\;\times \;100\%$$

### 2.8. Statistical Analysis

All determinations were carried out in triplicate and all statistical analysis was performed by means of OriginPro 2021.

## 3. Results and Discussion

### 3.1. Formation and Characterization of Oleogel

**Figure 2:**
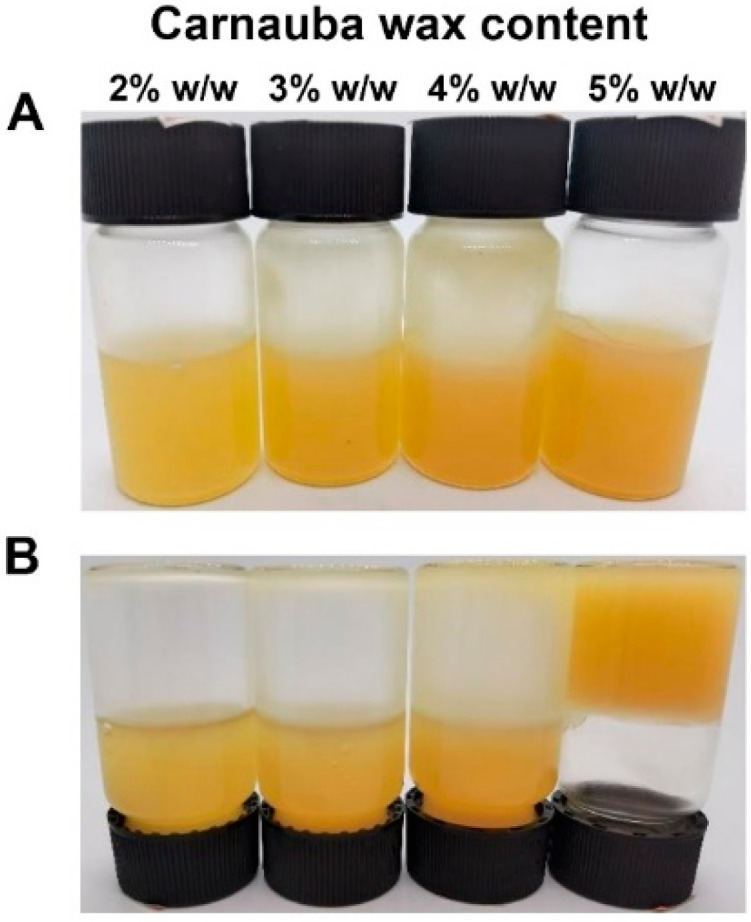
(**A**) Photographs of rice bran oil samples at different contents of carnauba wax (2–5% *w*/*w*); (**B**) Photographs of inverted rice bran oil samples at different contents of carnauba wax (2–5% *w*/*w*) after inverting the vials for 1 h.

Various contents of carnauba wax were mixed with rice bran oil to identify the critical gelling concentration. As shown in Figure 2, the obtained rice bran oil samples could flow at the carnauba wax concentration of 2% *w*/*w*, 3% *w*/*w* and 4% *w*/*w*, whereas the food-grade oleogel was successfully obtained at the carnauba wax concentration of 5% *w*/*w* because of immobility at room temperature. The formation of oleogel was attributed to crystallization as well as the coalescence of lipids, thereby restricting the movement of rice bran oil as the conventional high-melting-point solid fats [3]. Therefore, 5% *w*/*w* was regarded as the critical gelling concentration of carnauba wax-based oleogel and the oleogel was applied to the subsequent experiments in this research.

### 3.2. Formation and Characterization of OVA–GA Conjugate (OGC)

#### 3.2.1. Sodium Dodecyl Sulfate Polyacrylamide Gel Electrophoresis (SDS-PAGE)

**Figure 3:**
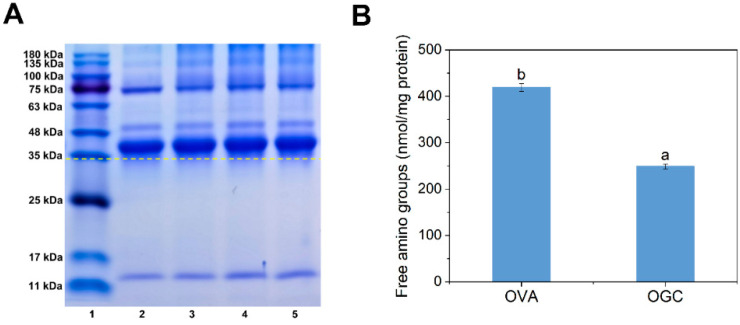
(**A**) SDS-PAGE profiles of OVA and OGC with different heat treatment times (24 h, 48 h and 96 h). Lane 1, protein marker; lane 2, OVA; lane 3, OGC heated for 24 h; lane 4, OGC heated for 48 h; lane 5, OGC heated for 96 h; (**B**) Free amino groups of OVA control and OGC heated for 96 h. Mean values (*n* = 3) with different lowercases represented significant differences (*p* < 0.05).

After the fabrication of OGC by means of a controlled dry heating method, SDS-PAGE analysis was performed in order to confirm the formation of covalent linking and to measure the molecular weight changes of the samples [28]. As depicted in Figure 3A, OGC with different heat treatment times of 24 h (lane 3), 48 h (lane 4) and 96 h (lane 5) exhibited upward bands compared with unmodified OVA (lane 2), implying the successful fabrication of OGC via Maillard reaction [29]. Moreover, the increased diffusion of the bands for OGC (lane three, lane four and lane five) was possibly due to the complex covalent OGC that one protein molecule was grafted with more than one polysaccharide [30]. Furthermore, a positive correlation was found between dry heating time and glycation extent, since the longer dry heating time of 96 h (lane 5) endowed the OGC with the significantly larger molecular weight of the protein compared with the shorter dry heating times of 24 h (lane 3) and 48 h (lane 4). Therefore, the relatively long dry heating time was conducive to the process of Maillard reaction and the fabrication of OGC.

#### 3.2.2. Free Amino Groups

Considering that the above SDS-PAGE analysis indicated that OGC heated for 96 h had a higher glycation extent than OGC heated for 24 h or 48 h, OGC heated for 96 h was chosen for the rest of this study. The amounts of free amino groups of OGC and OVA control were measured in order to identify the involvement of free amino groups in covalent linking between free amino acids of OVA and the carbonyl group of GA, thereby evaluating the glycation extent of OVA [31]. As shown in Figure 3B, the number of free amino groups in OGC dropped by 40.59% compared with that of OVA. The reduction of free amino groups was ascribed to the Maillard reaction between the free amino groups of the OVA and the reducing carbonyl groups of GA [32]. Since the amino acid residues on the protein surface or specific glycation “hot-spots” had priority at glycation modification [33], the surface and specific sites of OVA were glycated by dry heating treatment. The reaction sites of OVA including lysine residues and a small portion of arginine residues were grafted with the carbonyl group during the initial stages of the Maillard reaction [34]. It should be pointed out that the free amino groups of OVA included not only ε-NH_2_ of 15 arginine residues and 20 lysine residues but also α-NH_2_ in the N-terminus, which was favorable for the process of the Maillard reaction [35].

#### 3.2.3. Zeta Potential

**Figure 4:**
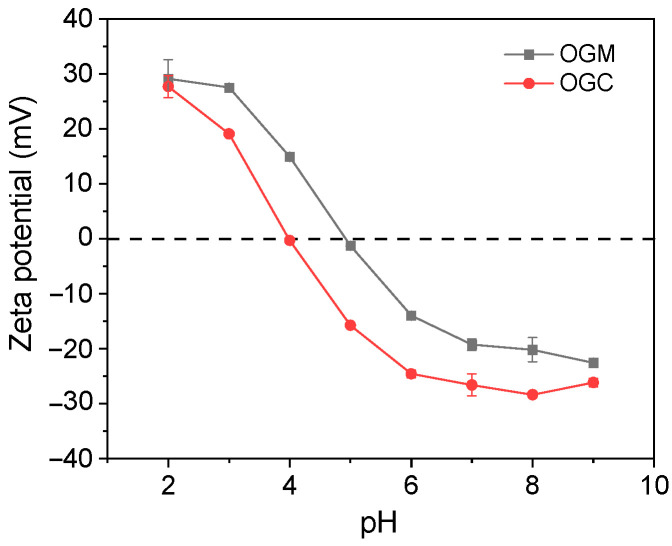
Zeta potential of OGM and OGC heated for 96 h.

The zeta potential of OGM and OGC was measured to explore the influence of the Maillard reaction on the surface charge of OVA and the change of OVA conformation [36]. Figure 4 shows the zeta potential as a function of pH. The OGC presented a left shift (from around pH 5.0 to pH 4.0) in the isoelectric point compared with OGM due to the Maillard reaction. There are two possible explanations for the result. On one hand, more negatively charged amino groups (such as –OH and –COOH) were exposed to the environment by the Maillard reaction [37]. On the other hand, the blocking of positively charged amino groups might also be the reason for the decrease of the isoelectric point according to the previous results [26]. According to the results of the free amino groups, it could be speculated that the latter was an essential factor in explaining the experimental phenomenon of the decreased isoelectric point. As indicated by the results of zeta potential measurement, pH 2 was chosen to serve as the satisfactory environment to fabricate oleogel-based nanoemulsions because OGC had the relatively high zeta potential at pH 2.

### 3.3. Formation and Characterization of Oleogel-Based Nanoemulsions

#### 3.3.1. Droplet Size of Oleogel-Based Nanoemulsions

**Figure 5:**
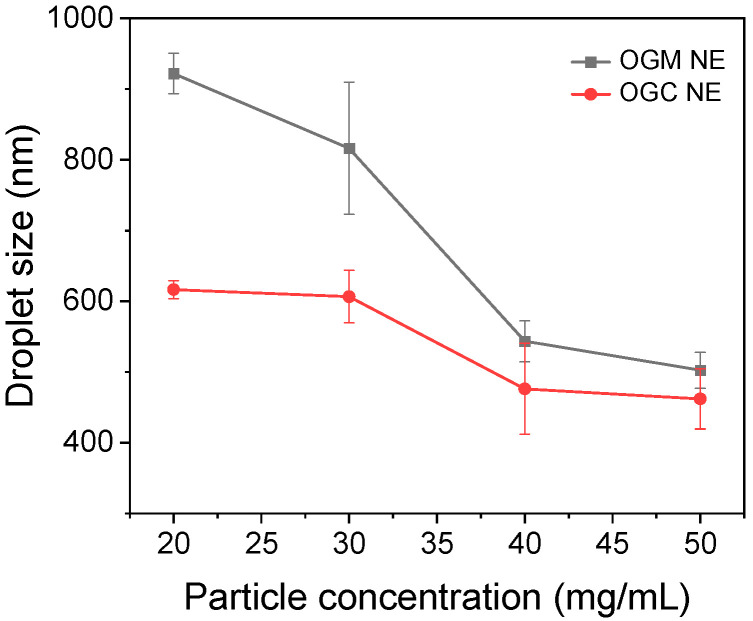
Average droplet size of freshly prepared OGM-stabilized oleogel-based nanoemulsions (NE) and the OGC-stabilized oleogel-based NE (oil fraction φ = 0.9) at different particle concentrations (20–50 mg/mL) at room temperature.

Given that high-internal phase emulsions (HIPEs) with an oil fraction (*φ*) above 0.74 possessed high nutraceutical loading [38], the oleogel-based nanoemulsions (*φ* = 0.9) were designed and prepared in this research. Nanoemulsions are the non-equilibrium emulsion systems with mean droplet size between 50 and 1000 nm [39]. The droplet sizes of oleogel-based nanoemulsions at different particle concentrations (20–50 mg/mL) were measured to compare OGM-stabilized nanoemulsions and OGC-stabilized nanoemulsions, thereby investigating the effect of the Maillard reaction on the interfacial properties of reaction products. As shown in Figure 5, the droplet sizes of the two oleogel-based nanoemulsions both exhibited a significant decrease with the increase of particle concentrations from 20 mg/mL to 50 mg/mL, which was ascribed to the decline of interfacial tension as the emulsifier concentration increased [40]. Moreover, the OGC-stabilized nanoemulsions had smaller emulsion droplets than OGM-stabilized nanoemulsions at each particle concentration under the same condition, indicating that the Maillard reaction endowed the oleogel-based nanoemulsions with superior emulsifying capability compared to physical mixing. This phenomenon could be explained as follows. First, the exposure of hydrophobic groups in OVA because of the Maillard reaction led to their enhanced affinity for the oil phase. Second, the extension of the hydrophilic carbohydrate moieties into the aqueous phase could hinder droplet aggregation via electrostatic repulsion or steric hindrance [41]. Third, the flexibility of OVA such as the conformational rearrangement of the tertiary protein structure possibly increased due to the Maillard reaction, which might promote the adsorption of OGC at the water-oil interface [42]. As depicted in Figures S1 and S2, the particle size distributions of OGM-stabilized nanoemulsions were broad, while that of OGC-stabilized nanoemulsions were relatively narrow. Consequently, Maillard-type protein–polysaccharide conjugates exhibited relatively desirable interfacial properties compared with electrostatic protein–polysaccharide complexes.

#### 3.3.2. Storage Stability of Oleogel-Based Nanoemulsions

**Figure 6:**
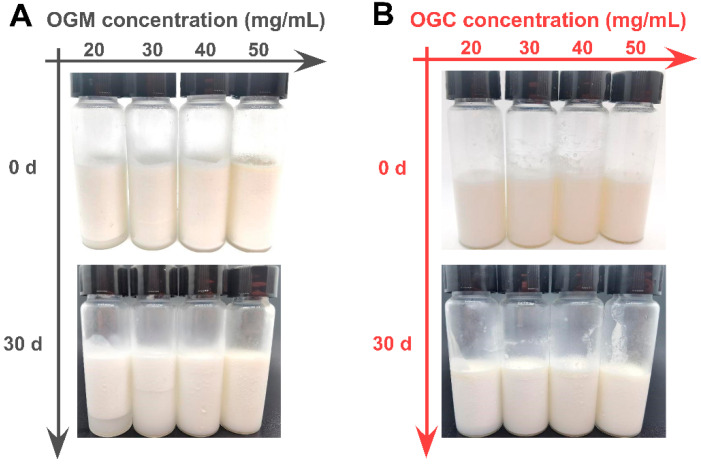
(**A**) Visual observation of oleogel-based nanoemulsions (oil fraction φ = 0.9) at different OGM concentrations (20–50 mg/mL) before and after 30 days of storage at 4 °C; (**B**) Visual observation of oleogel-based nanoemulsions at different OGC concentrations (20–50 mg/mL) before and after 30 days of storage at 4 °C.

In addition to the droplet size analysis of oleogel-based nanoemulsions, their visual observation was performed to further investigate the emulsifying properties of Maillard-type protein–polysaccharide conjugates and electrostatic protein–polysaccharide complexes. Figure 6 shows that the oleogel-based nanoemulsions at relatively low OGM concentration (20 mg/mL and 30 mg/mL) exhibited an obvious creaming phenomenon. Although the higher OGM concentration of 40 mg/mL could effectively alleviate the problem, the oleogel-based nanoemulsion at the OGM concentration of 50 mg/mL displayed an oiling-out behavior. On the contrary, the oleogel-based nanoemulsions at different OGC concentrations varying from 20 mg/mL to 50 mg/mL had no serum phase, implying better emulsifying properties than OGM. Since storage stability was an important indicator to evaluate the emulsion quality [43], the visual images of oleogel-based nanoemulsions after 30 days of storage at 4 °C were recorded as shown in Figure 6. Regrettably, different heights of bottom serum layer in the oleogel-based nanoemulsions at lower OGM concentrations (c = 20–40 mg/mL) were observed after 30 days of storage, indicating that the phase separation phenomenon became more apparent. It was noteworthy that the turbidity of the serum layer increased with the rise of OGM concentration, which might be ascribed to increased OGM concentration in the serum layer [44]. With regard to OGC-stabilized nanoemulsions, no serum layer emerged during storage. Combined with the results of droplet size analysis, the OGC-stabilized nanoemulsions with smaller droplets possessed stronger and more cohesive interfacial membranes, leading to superior stability against film fracture and gravitational separation. Meanwhile, the low frequency and rate of inter-droplet collision at 4 °C resulted in the decline of droplet size growth [45]. But one drawback that arose during storage was the slight oiling-out behavior of the oleogel-based nanoemulsions at lower OGC concentrations (c = 20–40 mg/mL). Whereas the relatively high OGC concentration of 50 mg/mL effectively alleviated the problem, implying that enough OGC concentration was significantly conducive to desirable storage stability of oleogel-based nanoemulsions for application in the food industry. Accordingly, Maillard-type protein–polysaccharide conjugates displayed better performance to stabilize oleogel-based nanoemulsions than electrostatic protein–polysaccharide complexes.

### 3.4. Lipolysis and Bioaccessibility of Astaxanthin in Oleogel-Based Nanoemulsions

**Figure 7:**
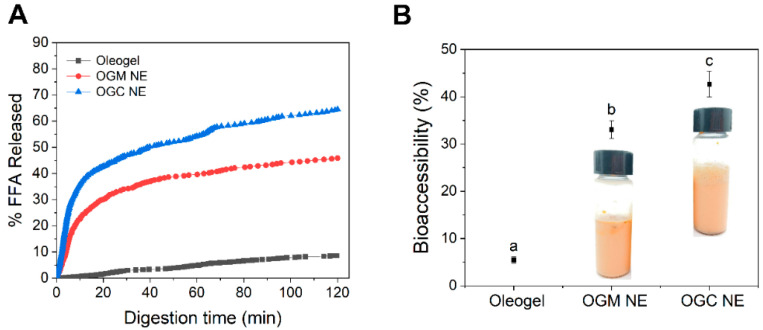
(**A**) Release profile of free fatty acids in oleogel, the OGM-stabilized oleogel-based nanoemulsions (NE), and the OGC-stabilized oleogel-based NE; (**B**) The bioaccessibility of astaxanthin in oleogel, the oleogel-based nanoemulsions containing OGM and the oleogel-based nanoemulsions containing OGC after in vitro digestion. The insets show the visual observation of the corresponding astaxanthin-loaded oleogel-based nanoemulsions. Mean values (*n* = 3) with different lowercases represented significant differences (*p* < 0.05).

In view of the fact that emulsion droplets with a relatively small size and a large surface area could facilitate the rapid release of nutraceuticals in the gastrointestinal tract [7], it could be speculated that an OGC-stabilized nanoemulsion with a smaller droplet size had a better nutraceutical delivery performance than the OGM-stabilized nanoemulsion. Figure 7A shows that the fractions of free fatty acids (FFA) released in both OGC-stabilized nanoemulsion (64.5%) and OGM-stabilized nanoemulsion (45.9%) were higher than that in oleogel (8.6%). The extent of lipolysis for OGC-stabilized nanoemulsion with relatively small emulsion droplets was significantly enhanced because of the increased contact area between the pancreatin and the triglycerides [46]. Moreover, the rate of lipolysis rapidly rose during the initial stages of in vitro intestinal digestion but gradually decreased during the digestion for both oleogel-based nanoemulsions. This phenomenon was ascribed to the aggregation of the digestion products and the reduced ratio of enzymes to oil [47].

In vitro bioaccessibility was measured to investigate the nutraceutical delivery performance of oleogel-based nanoemulsions. As depicted in Figure 7B, the astaxanthin bioaccessibility of either OGC-stabilized nanoemulsion (42.7% ± 2.7%) or OGM-stabilized nanoemulsion (33.1% ± 1.9%) was higher than that of oleogel (5.5% ± 0.7%), indicating that the oleogel-based nanoemulsions displayed outstanding nutraceutical delivery performance. Given that FFA promoted the formation of mixed micelles and the micelles could be used to solubilize astaxanthin, the release of astaxanthin could be regulated through the controlled lipolysis of oleogel-based nanoemulsions [48]. The structural destruction of oleogel-based nanoemulsions led to the exposure of the astaxanthin hydrophobic core, thereby contributing to the transfer of astaxanthin into the mixed micelles and improving astaxanthin bioaccessibility [49]. In addition, contrary to an inhomogeneous appearance of OGM-stabilized nanoemulsions, the OGC-stabilized nanoemulsion was homogeneous on the whole. This phenomenon indicated that OGC was a desirable emulsifier to stabilize oleogel-based nanoemulsions for nutraceutical delivery when compared with OGM. Furthermore, Figure 7 shows that the astaxanthin bioaccessibility positively correlated with both the extent of lipolysis and the visual observation of oleogel-based nanoemulsions. This result systematically demonstrated that oleogel-based nanoemulsions with outstanding nutraceutical delivery performance were successfully fabricated by Maillard-type OVA–GA conjugate in this study.

## 4. Conclusions

In summary, the OVA–GA conjugate (OGC) and the OVA–GA mixture (OGM) were prepared to stabilize food-grade oleogel-based nanoemulsions. The comparisons between electrostatic protein–polysaccharide complexes and Maillard-type protein–polysaccharide conjugates were subsequently performed. As indicated by the results, the OGC-stabilized nanoemulsions had smaller emulsion droplets and superior storage stability than OGM-stabilized nanoemulsions, indicating that OGC had more desirable emulsifying capability to stabilize oleogel-based nanoemulsions than OGM. The astaxanthin-loaded OGC-stabilized nanoemulsions, which could substantially improve the extent of lipolysis and astaxanthin bioaccessibility, exhibited a relatively homogeneous appearance, unlike the astaxanthin-loaded OGM-stabilized nanoemulsions. The outstanding nutraceutical delivery performance may endow OGC-stabilized oleogel-based nanoemulsions with great potential in the application of functional foods. This research focusing on the comparisons between electrostatic protein–polysaccharide complexes and Maillard-type protein–polysaccharide conjugates may also offer insights to the design, characterization and application of protein–polysaccharide complexes.

## Data Availability

The data presented in this study are available on request from the corresponding author.

## Supplementary Materials

The following are available online at https://www.mdpi.com/article/10.3390/foods11131859/s1, Figure S1. Particle size distribution of OGM-stabilized oleogel-based nanoemulsions at different OGM concentration. Figure S2. Particle size distribution of OGC-stabilized oleogel-based nanoemulsions at different OGC concentration.
