# Supplementary material for: Modulation of Fabrication and Nutraceutical Delivery Performance of Ovalbumin-Stabilized Oleogel-Based Nanoemulsions via Complexation with Gum Arabic
Source: Foods. 2022 Jun 24;11(13):1859. doi: 10.3390/foods11131859 (PMC9265802; doi:10.3390/foods11131859)
Supplement: Supplementary file 1 [file foods-11-01859-s001.zip › foods-1733562-supplementary.pdf]

foods

Supplementary Information
for

Modulation of Fabrication and Nutraceutical Delivery Performance of Ovalbumin-Stabilized Oleogel-Based Nanoemulsions via Complexation with Gum Arabic

Yuxing Gao [1], Zihua Wang [1], Changhu Xue [1,2], Zihao Wei [1,*]

[1] College of Food Science and Engineering, Ocean University of China, Qingdao 266003, China gaoyuxing@stu.ouc.edu.cn (Yuxing Gao); wangzihua@stu.ouc.edu.cn (Zihua Wang); xuech@ouc.edu.cn (Changhu Xue)
[2] Laboratory for Marine Drugs and Bioproducts, Qingdao National Laboratory for Marine Science and Technology, Qingdao 266237, China
* Correspondence: weizihao@ouc.edu.cn

Citation: Gao, Y.; Wang, Z.; Xue, C.; Wei, Z. Modulation of Fabrication and Nutraceutical Delivery Performance of Ovalbumin-Stabilized Oleogel-Based Nanoemulsions via Complexation with Gum Arabic. *Foods* **2022**, *11*, 1859. https://doi.org/10.3390/foods11131859

Academic Editor: Francesca Cuomo

Received: 4 May 2022
Accepted: 22 June 2022
Published: 24 June 2022

Publisher's Note: MDPI stays neutral with regard to jurisdictional claims in published maps and institutional affiliations.

Copyright: © 2022 by the authors. Submitted for possible open access publication under the terms and conditions of the Creative Commons Attribution (CC BY) license (https://creativecommons.org/licenses/by/4.0/).

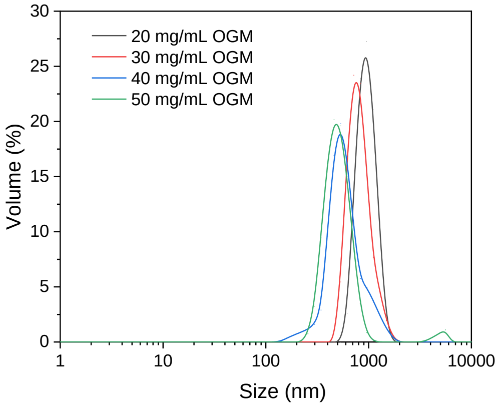

Figure S1. Particle size distribution of OGM-stabilized oleogel-based nanoemulsions at different OGM concentration.

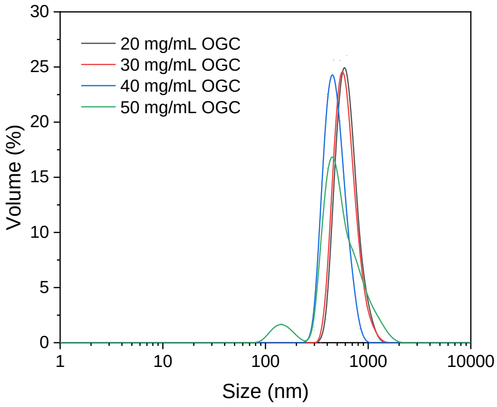

Figure S2. Particle size distribution of OGC-stabilized oleogel-based nanoemulsions at different OGC concentration.
